# HIV-1 inhibitory properties of eCD4-Igmim2 determined using an Env-mediated membrane fusion assay

**DOI:** 10.1371/journal.pone.0206365

**Published:** 2018-10-25

**Authors:** Edward Yang, Matthew R. Gardner, Amber S. Zhou, Michael Farzan, Ann M. Arvin, Stefan L. Oliver

**Affiliations:** 1 Departments of Pediatrics and Microbiology & Immunology, Stanford University School of Medicine, Stanford, California, United States of America; 2 Department of Infectious Diseases, The Scripps Research Institute, Jupiter, Florida, United States of America; "INSERM", FRANCE

## Abstract

Human Immunodeficiency Virus-1 (HIV-1) entry is dependent on the envelope glycoprotein (Env) that is present on the virion and facilitates fusion between the envelope and the cellular membrane. The protein consists of two subunits, gp120 and gp41, with the former required for binding the CD4 receptor and either the CXCR4 or CCR5 coreceptor, and the latter for mediating fusion. The requirement of fusion for infection has made Env an attractive target for HIV therapy development and led to the FDA approval of enfuvirtide, a fusion inhibitor. Continued development of entry inhibitors is warranted because enfuvirtide resistant HIV-1 strains have emerged. In this study, a novel HIV-1 fusion assay was validated using neutralizing antibodies and then used to investigate the mechanism of action of eCD4-Ig^mim2^, an HIV-1 inhibitor proposed to cooperatively bind the CD4 binding site and the sulfotyrosine-binding pocket of gp120. Greater reduction in fusion levels was observed with eCD4-Ig^mim2^ in the fusion assay than all of the gp120 antibodies evaluated. Lab adapted isolates, HIV-1_HXB2_ and HIV-1_YU2_, were sensitive to eCD4-Ig^mim2^ in the fusion assay, while primary isolates, HIV-1_BG505_ and HIV-1_ZM651_ were resistant. These results correlated with greater IC_50_ values for primary isolates compared to the lab adapted isolates observed in a virus neutralization assay. Analysis of gp120 models identified differences in the V1 and V2 domains that are associated with eCD4-Ig^mim2^ sensitivity. This study highlights the use of a fusion assay to identify key areas for improving the potency of eCD4-Ig^mim2^.

## Introduction

Human Immunodeficiency Virus type 1 (HIV-1) is the causative agent of acquired immunodeficiency syndrome (AIDS) [1]. Fusion of the HIV-1 virion envelope and the cell membrane is required for virus entry during infection [1]. This critical step in entry is mediated by HIV-1 envelope glycoprotein (Env), a class I fusogen that is expressed and cleaved into the mature glycoprotein 41 (gp41) and glycoprotein 120 (gp120) subunits in the Golgi prior to its incorporation into the virion envelope [2]. The gp120 subunit consists of five variable domains (V1 –V5) with the CD4 binding loop (CD4BL) present between the V3 and V4 domains [1,3]. Env membrane fusion is triggered via interaction of gp120 with the primary cellular receptor CD4 in conjunction with one or both of the chemokine receptors, CXCR4 or CCR5, which also serve as coreceptors [1]. This interaction facilitates a conformation change in gp41 which initiates membrane fusion [1]. The critical role of Env for entry has made the glycoprotein an attractive target for HIV treatment and led to the development and FDA approval of enfuvirtide, a gp41-binding fusion inhibitor [4]. While the inhibitor has been successful in limiting HIV-1 infection, the emergence of primary HIV isolates resistant to enfuvirtide in monotherapies emphasizes the need for new entry inhibitors [5].

The recently developed eCD4-Ig^mim2^ inhibitor has been demonstrated to neutralize a variety of HIV-1 isolates from various clades in cell culture and protect rhesus macaques from Simian/Human Immunodeficiency Virus (SHIV) infection [6]. The inhibitor consists of CD4-Ig, an immunoadhesion form containing CD4 domains 1 and 2, and a CCR5-mimetic sulfopeptide at the carboxyl-terminus of the IgG1 Fc domain. The inhibitor is proposed to cooperatively bind the CD4 receptor binding site of gp120, which includes the CD4BL and the CCR5 binding site located at the base of the V3 domain. The inhibitor was shown to have activity against a complete breadth of all HIV-1, HIV-2 and SIV isolates presumably because of the conservation of the receptor binding sites. While eCD4-Ig^mim2^ was engineered to bind gp120 and neutralize infection, its ability to inhibit Env mediated fusion by direct or indirect means has not been determined.

The HIV-1 envelope-cellular membrane fusion has been successfully modeled using cell-cell fusion assays to evaluate small molecules for HIV-1 entry inhibition properties prior to validation with infection studies using pseudotyped viruses [4]. Many of these assays rely on enumeration of fused cells, a labor-intensive process with high variability. The stable reporter fusion assay (SRFA) is a quantifiable and functional cell-cell fusion assay that addresses this limitation and has been previously adapted to model varicella zoster virus (VZV) and human endogenous retrovirus glycoprotein dependent fusion [7]. In this assay, effector cells that transiently express the viral glycoproteins are co-cultured with target cells that express the receptors required for fusion. Fusion between the cells results in a mixing of the cytoplasm of the two cells and the association of the reporter proteins, dual split protein-1 and- 2 [8]. Fusion is quantified by measuring either the reconstituted GFP or *renilla* luciferase activity. The assay has been adapted to determine the mechanism of action for neutralizing antibodies and identify receptors or coreceptors for viral fusogens [7,9].

In this study, gp120 domains that had a direct or indirect role in Env mediated fusion were identified by evaluating human monoclonal antibodies in the SRFA adapted to model HIV-1 membrane fusion. The CD4 binding loop of gp120 was further studied by evaluating the eCD4-Ig^mim2^ inhibitor in the SRFA using Env from lab adapted and primary isolates. Sensitivity to eCD4-Ig^mim2^ fusion inhibition in the SRFA and neutralization was found to differ among the isolates and postulated to be attributed to structural differences in V1/V2 of gp120.

## Materials and methods

### Cells

Chinese Hamster Ovary K1 (CHO) and CHO-DSP1 cells, which were generated in a previous study [7], were propagated using F-12K Nutrient Mixture with Kaighn's modification (Invitrogen) supplemented with 10% fetal bovine serum (FBS; Invitrogen) and penicillin (100 U/mL; Invitrogen) with CHO-DSP1 cells maintained under puromycin selection (8 μg/mL; Invitrogen). 293T, HeLa.CD4 (obtained through the NIH AIDS Reagent Program, Division of AIDS, NIAID, NIH: HeLa CD4 (HT4-6C) from Dr. Bruce Chesebro) [10,11], and HeLa.CD4-DSP2 (generated in this study) cells were propagated using DMEM with 4.5 g/L glucose, L-glutamine, and sodium pyruvate (Invitrogen) supplemented with 10% FBS and penicillin with the latter cell line maintained under puromycin selection (5 μg/mL). HEK293T cells (ATCC) used for generating pseudoviruses were grown in DMEM (Corning) supplemented with 10% FBS. The TZM-bl cells (obtained through the NIH AIDS Reagent Program, Division of AIDS, NIAID, NIH: TZM-bl from Dr. John C. Kappes, Dr. Xiaoyun Wu and Tranzyme Inc) [5,12–15] were maintained in the recommended propagation medium provided in the specification sheet.

### Antibodies

The following antibodies were obtained from the NIH AIDS Reagent Program, Division of AIDS, NIAID, NIH: 17b (4091) from Dr. James E. Robinson [3,16–20], 2G12 (1476) from Dr. Hermann Katinger [21–25], 48d (1756) from Dr. James Robinson [16], 697-30d (7371) from Dr. Susan Zolla-Pazner [26–28], VRC03 (12032) from Dr. John Mascola [29], 71–31 (530) from Dr. Susan Zolla-Pazner [30].

### Vectors

The following vectors were obtained from the NIH AIDS Reagent Program, Division of AIDS, NIAID, NIH: pHXB2-env from Dr. Kathleen Page and Dr. Dan Littman [31], pCMV-rev from Dr. Marie-Louise Hammarskjöld and Dr. David Rekosh [32], pc-CCR5 from Dr. Nathaniel Landau [33,34]. The pCAGGS-SF2env vector was generated by PCR using primers EcoR1+KS-SF2 Env_F (5’-CGGGAATTCGCCATGAAAGTGAAGGGGACCAG-3’)and Xho1-SF2 Env_R (5’-ACGCTCGAGTTATAGCAAAAGCCTTTCCA-3’) to amplify HIV-Env(SF2) from the pCB-34 plasmid (gift from Dr. Edward Berger) [35]. The PCR product and the pCAGGs plasmid were digested with EcoR1 and Xho1 and then ligated together with T4 ligase. The plasmid was confirmed by restriction digest and sequencing. The pZM651-env plasmid is a codon optimized ZM651 Env cloned into the pcDNA3 expression vector.

### Construction of HeLa.CD4-DSP2

The HeLa.CD4-DSP2 cells were generated using a protocol adapted from a previous study [7]. Briefly, lentiviruses were generated by transfecting 293T cells using pGIPZ-DSP2, psPAX2 and pMD2.G plasmids using Lipofectamine 2000 (Invitrogen). A spinoculation was performed to generate HeLa.CD4-DSP2 cells with puromycin (Invitrogen) added at 48 hours post transduction for selection.

### HIV-Env fusion assay

For the HIV-1_HXB2_ Env fusion assay, 8x10^5^ CHO-DSP1 cells were transfected with 0.25 μg each of pHXB2-env and pCMV-rev plasmids using Lipofectamine 2000 (Invitrogen). The HIV-1_HXB2_ Env protein is from an extensively characterized laboratory strain used in the development of HIV fusion inhibitors [36]. At six hours post transfection, the transfected CHO cells were trypsinized, centrifuged at 400 RCF for five minutes and resuspended in 1 mL media. Of the 1 mL, 125 μl were mixed with 3.33x10^5^ HeLa.CD4-DSP2 cells and seeded in 12-well plates. HeLa.CD4 cells were selected as the target cell for their ability to support HIV-1_HXB2_ infection [37–39]. At 24 hours post co-culture, cells were trypsinized, centrifuged at 400 RCF for five minutes and resuspended in 300 μl of resuspension buffer (PBS, 2.5% FBS, 5 mM EDTA). Of the 300 μl, 100 μl of resuspended cells was added to duplicate wells of a 96 well plate and incubated with membrane permeable colenterazine-H (5 μM, Nanolight Technology) substrate for five minutes at room temperature. Fusion levels were quantified by measuring luminescence (lumens) using a Synergy H1 Multi-mode reader (Biotek).

Both the monoclonal antibody and small peptide inhibition of HIV-1_HXB2_ Env fusion assays were performed using the 12-well format described above. For the monoclonal antibody fusion inhibition assay of HIV-1_HXB2_ Env, the co-cultures were incubated with 10 μg/mL of either anti-HIV gp120 antibodies, 17b, 2G12, 48d, 697-30d, or VRC03, or anti-HIV p24, 71–31. For the small peptide fusion inhibition assay, the co-cultures were incubated with serially diluted enfuvirtide (T-20) (9409, obtained through the NIH AIDS Reagent Program, Division of AIDS, NIAID, NIH: T-20, Fusion Inhibitor from AIDS, NIAID), eCD4-Ig^mim2^ [6], gE-Ig, and Ig at a concentration of 0.01, 0.1 or 1 μg/mL. Fusion levels were quantified as described above.

For the human serum HIV neutralization assay, CHO-DSP1 cells were transfected with 1 μg each of pCAGGs-SF2env and pCMV-rev plasmids. At six hours post transfection, the cells were trypsinized, centrifuged, and resuspended in 1 mL of media, of which 62.5 μl of transfected CHO cells were mixed with HeLa.CD4-DSP2 cells (3.98x10^5^) and seeded in 24-well plates. HIV-1 neutralizing reference serum 1 (1984) and 2 (1983) and HIV-1 negative control human serum (2411), which were obtained through the NIH AIDS Reagent Program, Division of AIDS, NIAID from Dr. Luba Vujcic, FDA, Center for Biologics Evaluation and Research, were added to the cells at a working dilution of 1:50, 1:100, and 1:200 [40]. At 24 hours post co-culture, cells were harvested and resuspended in 400 μl of resuspension buffer, of which 100 μl was added to triplicate wells of a 96 well plate with fusion levels quantified using the approach described above.

The small peptide inhibition of HIV-1_YU2_, HIV-1_BG505_, and HIV-1_ZM651_ Env fusion assay followed the approach described above for HIV-1_HXB2._ CHO-DSP1 cells were transfected with 0.75 μg of pYU2-env [6], 0.75 μg pCMV-rev and 0.15 μg pTet [6]; 2 μg each of pBG505-env (gift from Dr. John Moore and Dr. Per-Johan Klasse) and pCMV-rev; and 0.75 μg each of pZM651-env and pCMV-rev, respectively. A 100 mm-dish of 6x10^6^ HeLa.CD4-DSP2 cells was transfected with 24 μg of pc-CCR5 vector. At six hours post transfection, CHO-DSP1 cells were trypsinized, collected by centrifugation, resuspended in media and mixed with 3.33x10^5^ HeLa.CD4-DSP2 cells transiently expressing CCR5. Fusion levels were quantified at 24 hours post co-culture as described above. All experiments were performed at minimum in duplicate.

### Purification of Ig fusion peptides

Ig fusion peptides, eCD4-Ig^mim2^, CD4-Ig, gE-Ig, and Ig only were purified from 293T cells cotransfected with pcDM8-eCD4-Ig^mim2^, pFuse-IL2FCgE or pFuse-IL2FC, respectively, and pTpst2, which expresses tyrosylprotein sulfotransferase 2 as previously described [6].

### TZM-bl neutralization assay

Pseudotyped HIV-1 was produced by co-expression of envelope glycoproteins of YU2, ZM651, BG505 or HXB2 with NL4-3ΔEnv [6]. 293T cells were grown to 50% confluency in T175 (Falcon) flasks. Cells were transfected with 25 μg of plasmid encoding the envelope glycoprotein, 45 μg of NL4-3ΔEnv, and 5 μg each of HIV-1 tat and rev plasmids using a calcium phosphate transfection kit (Clontech). Medium was changed at 12 hours and collected at 48 hours post transfection. Viral supernatants were cleared by centrifugation for 10 min at 1500 RCF, passed through a 0.45-μm syringe filter (Millipore), and stored at -80°C. TZM-bl neutralization assays were performed as previously described [6]. Briefly, HIV-1 pseudoviruses were pre-incubated with titrated amounts of eCD4-Ig^mim2^ or CD4-Ig in DMEM (10% FBS) for 1 hour at 37°C. TZM-bl cells were detached by trypsinization and diluted in DMEM (10% FBS) to 1x10^5^ cells/mL. Diluted TZM-bl cells (100 μl) were added to the pseudovirus/inhibitor mixture. Cells were then incubated for 48 hours at 37°C. Viral entry was analyzed using Britelite Plus (Perkin Elmer) and luciferase expression was measured using a Victor X3 plate reader (Perkin Elmer). IC_50_ and IC_90_ values were determined for both inhibitors using a non-linear regression curve fit by GraphPad Prism 6.0.

### Sequence alignment

The amino acid sequence of HIV-1_HXB2_ (P04578), HIV-1_YU2_ (P35961), HIV-1_BG505_ (Q2N0S6), and HIV-1_ZM651_ (AAK30970.1) were aligned using Clustal Omega. (http://www.ebi.ac.uk/Tools/msa/clustalo/). The generated FASTA file from Clustal Omega was displayed using GeneDoc [41] and further annotated using Adobe Illustrator. The amino acid sequences were submitted to N-GlycoSite (https://www.hiv.lanl.gov/content/sequence/GLYCOSITE/glycosite.html) to predict potential N-linked glycosylation sites [42].

### Modeling of gp120 subunit structures

Molecular graphics and analyses were performed with the UCSF Chimera package [43]. Chimera is developed by the Resource for Biocomputing, Visualization, and Informatics at the University of California, San Francisco (supported by NIGMS P41-GM103311). Predicted models of the full length gp120 subunit of HIV-1_HXB2_, HIV-1_YU2,_ HIV-1_BG505_, and HIV-1_ZM651_ were generated by submitting the amino acid sequence to RaptorX [44]. The model with the lowest p-value was used for analysis and structure comparisons were made using MatchMaker (UCSF Chimera). To model binding of the 17b, 48d, and VRC03 Fabs as well as CD4 domains 1 and 2 to HIV-1_HXB2_ gp120, a structural comparison was performed using the homology model and the gp120 structure of HIV-1clade A/E 93TH0573SE8 complexed with VRC03 (3SE8) [45], HIV-1_YU2_ complexed with 17b Fab (4RQS) [46], and HIV-1_YU2_ complexed 48d Fab (4DVR) [47], HIV-1_YU2_ complexed with CD4 (2QAD) [48], respectively. The solved gp120 structures were removed to improve visualization.

### Statistical analysis

All quantitative results were analyzed by either t-test or one-way or two-way ANOVA to determine statistical significance using Prism (Graphpad Software).

## Results

### Validation of a novel HIV-1 fusion assay using human sera and neutralizing antibodies

**Fig 1:**
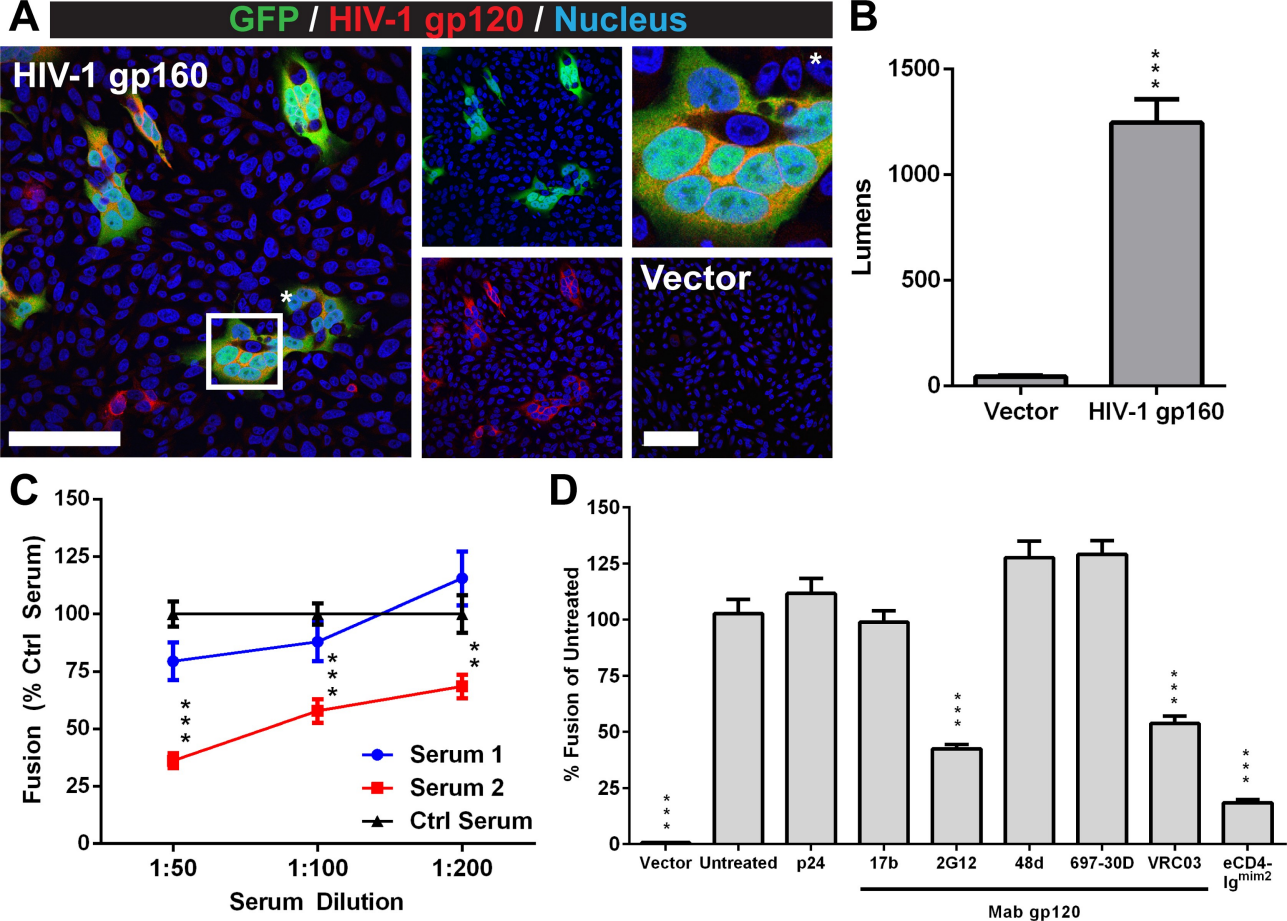
Quantification of antibody inhibition of HIV-1 Env fusion using the SRFA. (A) Confocal micrographs of CHO-DSP1 cells expressing HIV-1_HXB2_ Env and HeLa.CD4-DSP2 cells at 24 hours post co-culture. Cells were stained for HIV-gp120 (red) as a marker for Env and nuclei (Hoechst 33342; blue). Fused cells have GFP activity from reconstituted DSP1 and DSP2 (Green). Merged image is on the left. Middle images are of GFP and nuclei (top) and HIV-gp120 and nuclei (bottom). Right top image (*) is a magnification of a representative fused cell. Right bottom image is the vector control. The white scale bars represent 100 μm. (B) Fusion levels of HIV-1_HXB2_ Env quantified by luminescence (lumens) at 24 hours post co-culture. (C) HIV neutralizing serum inhibition of HIV-1_SF2_ Env fusion. Co-cultures of CHO-DSP1 cells transiently expressing HIV-1_SF2_ Env and Hela.CD4-DSP2 cells incubated with serially diluted serum from two HIV-1 asymptomatic infected patients (Serum1, blue; Serum2; red). A pooled serum from HIV noninfected individuals was used as the control (Ctrl Serum; black). Test samples were normalized and compared to the Ctrl Serum at their respective dilutions. (D) HIV-1_HXB2_ Env fusion inhibition properties of anti-gp120 monoclonal antibodies 17b, 2G12, 48d, 697-30D or VRC03 at 10 μg/mL or one μg/mL of eCD4-Ig^mim2^. Monoclonal antibody to HIV-1 p24 (mAb gp120) was used as a nonspecific IgG antibody control. Negative control (Vector) was cells transfected with empty vector. All test samples were normalized and compared to the untreated control (Untreated). Test samples were normalized and compared to the Ctrl Serum at their respective dilutions. Statistical analysis was performed using t-test (B) (***P<0.001) or ANOVA (C,D) (**P<0.01, ***P<0.001). Data shown represents the mean of at minimum two independent experiments with bars indicating the standard error of the mean (SEM).

The SRFA was adapted to model and quantify Env-mediated fusion using CHO cells transiently expressing Env from the CXCR4 tropic HIV-1_HXB2_ (clade B), a lab adapted isolate, and target HeLa cells expressing CXCR4 and CD4. Fused cells with reconstituted GFP activity and Env expression were observed at 24 hours post co-culture by confocal microscopy (Fig 1A). Fusion was quantified by measuring luminescence from the reconstituted *renilla* luciferase in the fused cells. Co-cultures expressing HIV-1_HXB2_ Env had a 27-fold increase in luminescence compared to the vector control (Fig 1B). Thus, HIV-1 Env mediated fusion can be modeled by the SRFA.

Sera from HIV patients can contain antibodies generated from a natural infection that target either gp41 or gp120 [49]. A subset of these antibodies would have Env fusion inhibitory properties. To demonstrate the capacity of the SRFA to characterize polyclonal sera for fusion inhibition properties, well-characterized reference sera obtained from two HIV-1 infected patients (Serum1 and Serum2) and pooled serum from four uninfected individuals (Ctrl Serum) were evaluated using the HIV-1_SF2_ Env, a CXCR4 tropic virus previous found to neutralize virus at 1:320 and 1:410 dilutions, respectively [40]. Serum2 reduced fusion in a concentration dependent manner with the 1:50 and 1:200 dilutions reducing fusion levels to 36% and 68% of the Ctrl Serum, respectively (Fig 1C). The reduction in fusion by Serum2 was observed at a higher concentration than the neutralization titer of 1:410 dilution previously determined by a virus neutralization assay [40]. The difference in fusion and virus neutralization titers was attributed to the transient expression of Env in the SRFA, which would generate higher copies of Env needed to be neutralized relative to the virus neutralization assays. In contrast to Serum2, fusion levels of cells incubated with Serum1 did not differ from the control, suggesting that Serum1 had lower levels of antibodies that could inhibit HIV-1_SF2_ Env fusion compared to Serum2. Thus, patient sera containing antibodies that directly or indirectly inhibit Env mediated fusion can be identified using the SRFA.

**Fig 2:**
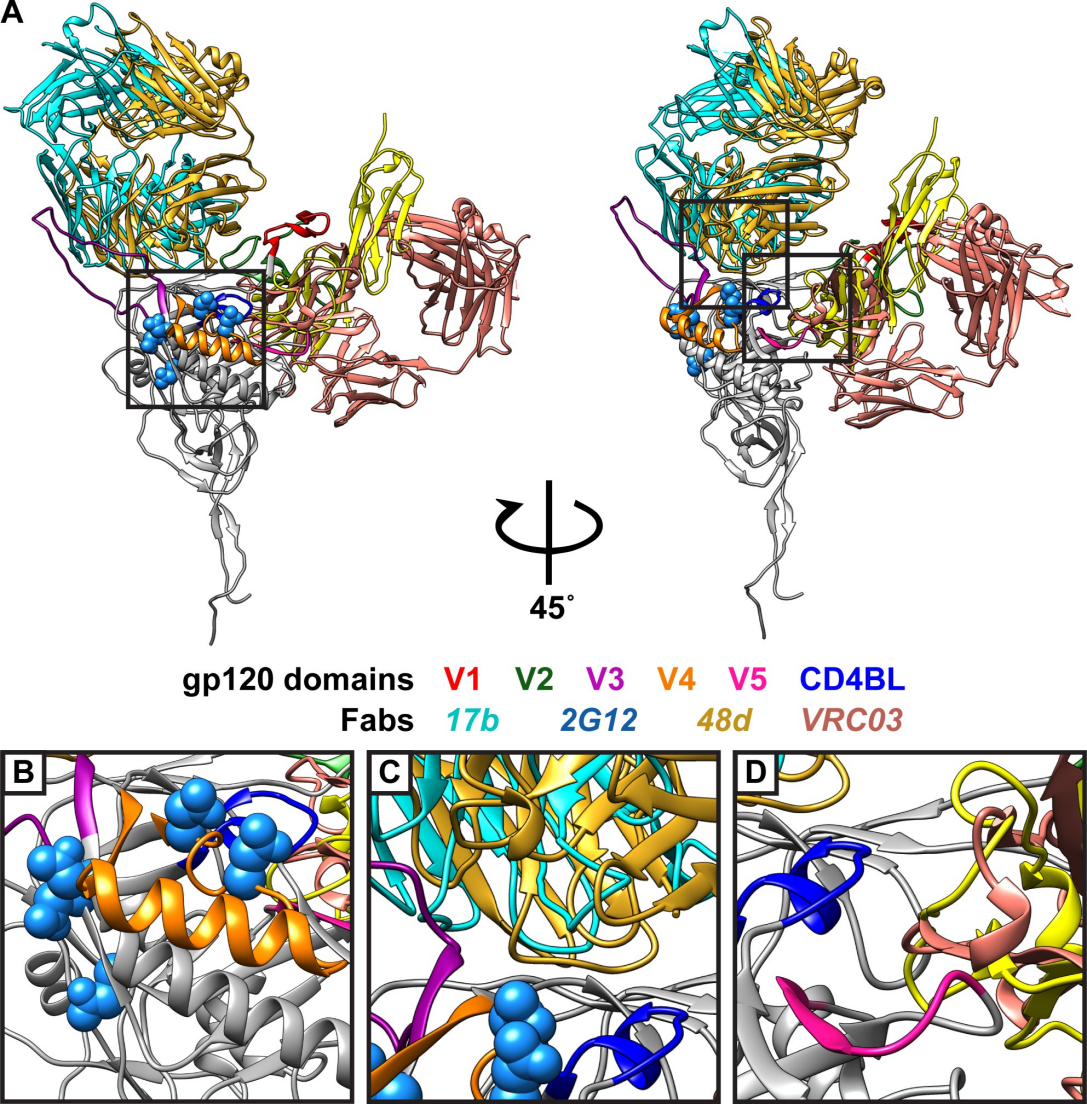
Structural homology model of HIV-1_HXB2_ gp120 bound to CD4 with human monoclonal antibodies 17b, 2G12, 48d, and VRC03. (A) Ribbon diagram of HIV-1_HXB2_ gp120 structural homology model (grey) bound with CD4 domains 1 and 2 (2QAD; yellow) generated by RaptorX with V1 (red), V2 (dark green), V3 (purple), V4 (orange), and V5 (pink) domains and the CD4 binding loop (CD4BL; blue). The image on the right represents the structure on the left rotated 45 degrees to the left. The boxed area of the left structure is magnified in (B) and highlights the residues required for 2G12 epitope recognition which are indicated by light blue spheres (N295, N332, N386, N392, and N448). The upper boxed area of the right structure is magnified in (C) and highlights the binding of the 17b (4RQS; cyan) and 48d (4DVR; gold) Fabs to their respective CD4i epitopes. The lower boxed area of the right structure is magnified in (D) and highlights the interaction of the VRC03 Fab (3SE8; light pink) and the CD4 binding site which contains the CD4BL. The VRC03 Fab also occupies the same space as where the CD4 domains 1 and 2 (yellow) interact with the gp120 subunit.

To expand on the serum neutralizing data, neutralizing monoclonal antibodies (mAbs) with known epitopes were used to identify domains of the fusion protein that are important for the entry process. The mAbs, 17b, 2G12, 48d, 697-30D and VRC03 were derived from HIV-1 infected patients and are known to bind the gp120 subunit [16,21,26,29] (Fig 1D). To determine their ability to inhibit HIV-1_HXB2_ Env fusion in the SRFA, the mAbs were tested at 10 μg/mL. The 2G12 mAb is broadly neutralizing and has an epitope dependent on the asparagine residues of the gp120 subunit with N-linked glycosylations at positions 295 and 332 that flank the V3 domain, 386 and 392 that are within the V4 domain, and 448 (HIV-1_HXB2_ numbering) [50] (Fig 2A and Fig 2B). The 2G12 antibody reduced fusion to 42% compared to the control (Untreated). This reduction correlated with previous observations of reduced syncytia in cells transiently expressing Env and neutralization of HIV-1_HXB2_ infection of HeLa cells expressing CD4 [21,51]. The 2G12 antibody has been previously determined to have an IC_50_ of 0.47 μg/mL using a pseudovirus neutralization assay, which indicated that the SRFA had a reduced sensitivity to inhibition compared to virus neutralization assays [52]. The VRC03 mAb, which recognizes an epitope that overlaps with the CD4BL [3] (Fig 2D), reduced fusion to 53% compared to the control (No Ab). While the fusion inhibitory properties have not been previously reported for VRC03, the antibody can broadly neutralize viruses pseudotyped with Env from HIV-1 clade B strains, which includes HIV-1_HXB2_ [29].

In contrast to the 2G12 and VRC03 mAbs, HIV-1_HXB2_ Env fusion in the SRFA was not reduced by gp120 mAbs 697-30D, 17b, 48d or the IgG control antibody that binds HIV p24, 71–31 (Fig 1C). The 697-30D mAb recognizes a conformational epitope in the V2 domain and while neutralization studies of 697-30D have not been performed with HIV-1_HXB2_, the absence of fusion reduction by the mAb was consistent with its failure to neutralize HIV-1_IIIb_, a lab adapted strain similar to HIV-1_HXB2_ [26]. Both the 17b and 48d mAbs bind to the CD4i epitope that is transiently exposed on the gp120 subunit upon CD4 binding (Fig 2C) [53]. The absence of fusion inhibition by 17b and 48d mAbs in the SRFA was consistent with previous observations using cell fusion/syncytia assays and correlated with their weak neutralization activity in cell culture [16,54,55]. Thus, the modified SRFA successfully models HIV Env fusion and can identify antibodies that can directly or indirectly inhibit fusion.

### Fusion mediated by HIV-1_HXB2_ Env is inhibited by eCD4-Ig^mim2^ in the SRFA

**Fig 3:**
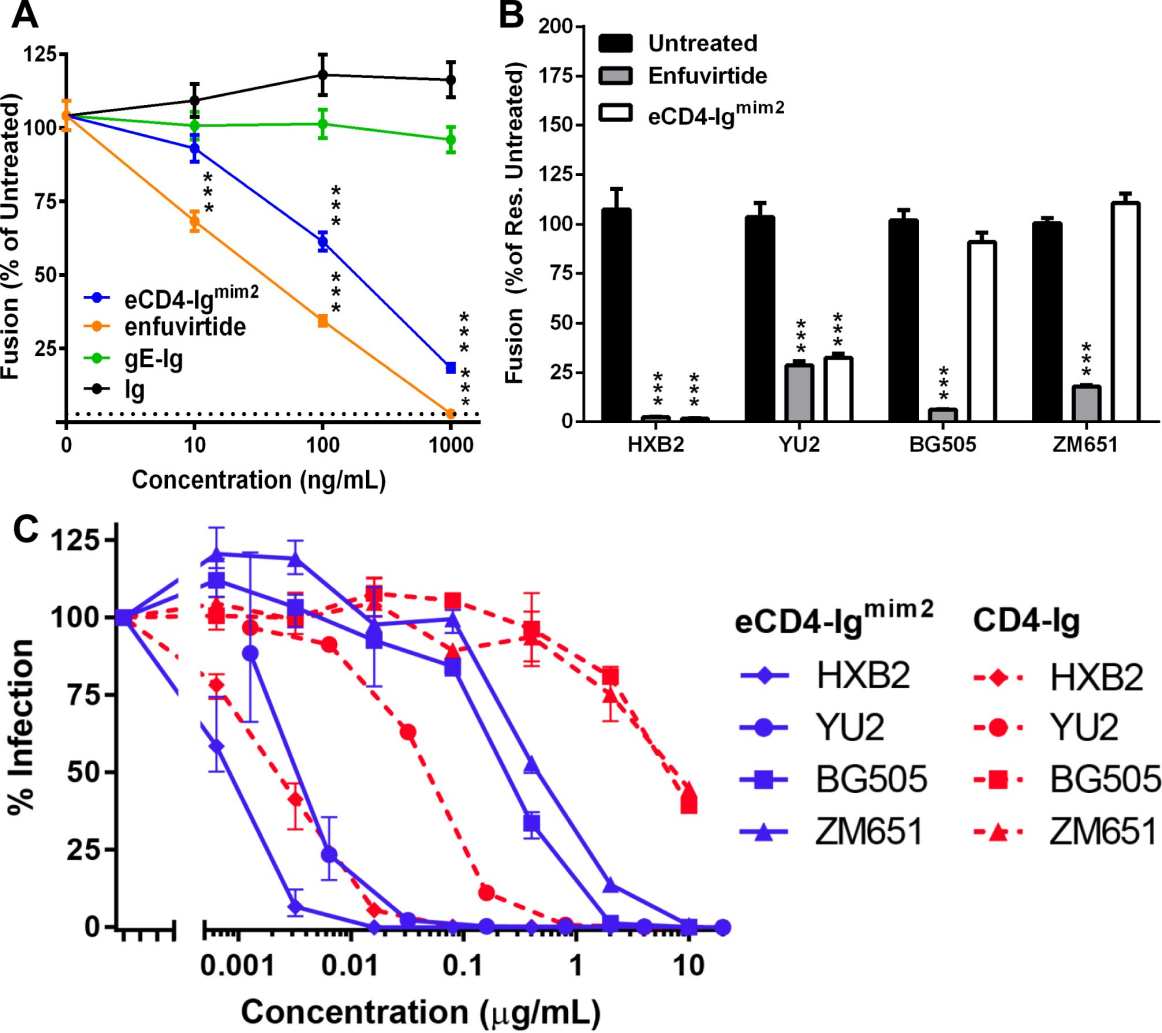
eCD4-Ig^mim2^ is less effective at neutralizing HIV-1_BG505_ and HIV-1_ZM651_ than HIV-1_YU2_ pseudotyped viruses. (A) Inhibition of HIV-1_HXB2_ Env fusion using equal titrated concentrations of eCD4-Ig^mim2^ (blue), enfuvirtide (orange), and the two control Igs, gE-Ig (green) and Ig only (Ig, black). Dotted line represents background levels of fusion from cells transfected with the empty vector control (Vector). All test samples normalized to the untreated cells (Untreated). (B) Inhibition of HIV-1_YU2_, HIV-1_BG505_, and HIV-1_ZM651_ Env fusion using equimolar concentrations (260nM) of eCD4-Ig^mim2^ (white) and enfuvirtide (grey). CHO-DSP1 cells transiently expressing the fusion proteins were co-cultured with Hela.CD4 cells transiently expressing CCR5. Test samples were normalized and compared to their respective untreated controls (black). Statistical analysis was performed using ANOVA (A and B)(***P<0.001). Data represent the mean for minimum of two independent experiments with bars indicating the standard error of the mean (SEM). (C) HIV-1 pseudotyped with Env from HXB2 (X4, clade B), YU2 (R5, clade B), ZM651 (R5, clade C), and BG505 (R5, clade A) isolates were pre-incubated for 1 hour with eCD4-Ig^mim2^ or CD4-Ig at varying concentrations starting at 10–20 μg/mL. TZM-bl cells were then added and incubated for 48 hours. Infection was measured by the percentage luciferase expression in the absence of inhibitor. Error bars indicate range of each experiment.

The reduction in fusion by the VRC03 mAb indicated the CD4BL of gp120 to be critical for fusion, making the domain an attractive drug target [3]. The eCD4-Ig^mim2^ inhibitor has been shown to be neutralizing and is thought to bind at the same site on gp120 as CD4, which includes the CD4BL [6]. To determine if eCD4-Ig^mim2^ was able to inhibit HIV-1 Env mediated fusion, the inhibitor was evaluated using the SRFA with HIV-1_HXB2_ Env (Fig 1D). The eCD4-Ig^mim2^ inhibited fusion at a concentration of one μg/mL compared to the untreated control with fusion levels reduced to 18% relative to the untreated control. This reduction in fusion by eCD4-Ig^mim2^ was greater than all of the antibodies tested in this study. The fusion inhibition properties of eCD4-Ig^mim2^ were then compared to enfuvirtide at three concentrations (10, 100, and 1000 ng/mL) (Fig 3A). Both eCD4-Ig^mim2^ and enfuvirtide reduced HIV-1_HXB2_ Env fusion in a concentration dependent manner. The fusion reduction was consistent with previous observations of enfuvirtide inhibition of HIV-1_HXB2_ pseudovirus entry [56]. The IC_50_ for enfuvirtide in the SRFA was roughly 12.5 nM, which was higher than the reported IC50 of 1.8 nM which was determined by a neutralization assay using lentivirus pseudotyped with HIV-1_HXB2_ Env [57]. This difference in IC50 between the two assays was attributed to the greater level of Env protein present in the SRFA compared to the neutralization assay. A difference in fusion reduction by eCD4-Ig^mim2^ and enfuvirtide at the 1000 ng/mL concentration was observed and initially attributed to the difference in molarity of 10 nM and 2200 nM, respectively. This was confirmed when HIV-1_HXB2_ Env fusion was reduced to ~2% of the untreated control for both eCD4-Ig^mim2^ and enfuvirtide tested at the equal molar concentration of 260 nM (Fig 3B). The similar reduction in fusion indicated that eCD4-Ig^mim2^ was as effective as enfuvirtide at inhibiting fusion by HIV-1_HXB2_ Env. Thus, HIV-1 neutralization by eCD4-Ig^mim2^ is a consequence of direct or indirect inhibition of Env mediated fusion.

While HIV-1 isolates resistant to broadly neutralizing antibodies have been shown to be susceptible to eCD4-Ig^mim2^ neutralization, the sensitivity to the inhibitor can vary as the IC_50_ values were found to range from 0.001 to 1.453 μg/mL [6]. To determine if fusion inhibition in the SRFA by eCD4-Ig^mim2^ could reveal the sensitivity of isolates to the inhibitor, Env from CCR5-tropic HIV isolates, HIV-1_YU2_ (clade B), and two untested isolates, HIV-1_BG505_ and HIV-1_ZM651_, were evaluated using HeLa target cells that transiently expressed CCR5 and stably expressed CD4 and CXCR4. In contrast to HIV-1_HXB2,_ all three viruses were primary isolates with HIV-1_BG505_ and HIV-1_ZM651_ used as prototypes for clades A and C, respectively. HIV-1_YU2_ Env fusion was reduced to ~30% of the untreated control by both eCD4-Ig^mim2^ and enfuvirtide at the 260 nM concentration, indicating that both are equally effective at inhibiting fusion by HIV-1_YU2_ Env (Fig 3B). However, while enfuvirtide reduced fusion of HIV-1_BG505_ and HIV-1_ZM651_ Env to 6% and 18% of their controls, respectively, eCD4-Ig^mim2^ did not affect fusion for either isolate. Thus, the eCD4-Ig^mim2^ was demonstrated to have reduced potency against HIV-1_BG505_ and HIV-1_ZM651_ compared to HIV-1_YU2_.

To determine if sensitivity to eCD4-Ig^mim2^ fusion inhibition in the SRFA correlated with HIV-1 neutralization, a TZM-bl neutralization assay was performed using eCD4-Ig^mim2^ and the immunoadhesion form of CD4 (CD4-Ig) which lacked the CCR5-mimetic sulfopeptide with viruses pseudotyped with HIV-1_HXB2_, HIV-1_YU2_, HIV-1_BG505_, and HIV-1_ZM651_ Env (Fig 3C). The eCD4-Ig^mim2^ inhibitor neutralized all four viruses with IC_50_ values ranging from <0.001 to 0.468 μg/mL (Table 1). The IC_50_ for HIV-1_YU2_ was 0.003 μg/mL, which was consistent with previous reports [6]. In contrast, eCD4-Ig^mim2^ was less effective at neutralizing HIV-1_BG505_ and HIV-1_ZM651_ than HIV-1_YU2_, with IC_50_ values that were 86 and 156 greater than HIV-1_YU2_, respectively. These results correlated with the lack of fusion inhibition observed in the SRFA. The HIV-1_HXB2_ was the most sensitive to the eCD4-Ig^mim2^ inhibitor of all the viruses tested with an IC_50_ that was 3.75 times less than HIV-1_YU2,_ which correlated with the fusion inhibition data and indicated that CXCR4 tropic viruses might be more sensitive to the inhibitor than CCR5 tropic viruses. The CD4-Ig inhibitor was also able to neutralize all four viruses, albeit at higher concentrations than eCD4-Ig^mim2^, which correlated with previously observed trends [6]. Thus, Env-mediated fusion inhibition in the SRFA correlated with the potency of eCD4-Ig^mim2^ against the specific HIV-1 isolate.

**Table 1:** The IC50 and IC90 values from eCD4-Ig^mim2^ and CD4-Ig neutralization of HIV-1 pseudoviruses.

	IC_50_(μg/mL)		IC_90_(μg/mL)	
	eCD4-Ig^mim2^	CD4-Ig	eCD4-Ig^mim2^	CD4-Ig
**HXB2**	**0.0008**	**0.002**	**0.002**	**0.008**
**YU2**	**0.003**	**0.06**	**0.009**	**0.165**
**BG505**	**0.258**	**4.66**	**0.83**	**>10**
**ZM651**	**0.468**	**7.54**	**1.65**	**>10**

### The eCD4-Ig^mim2^ resistant isolates have longer V2 domains and greater number of potential N-linked glycosylations within the V1 and V2 domains relative to the sensitive isolates

**Fig 4:**
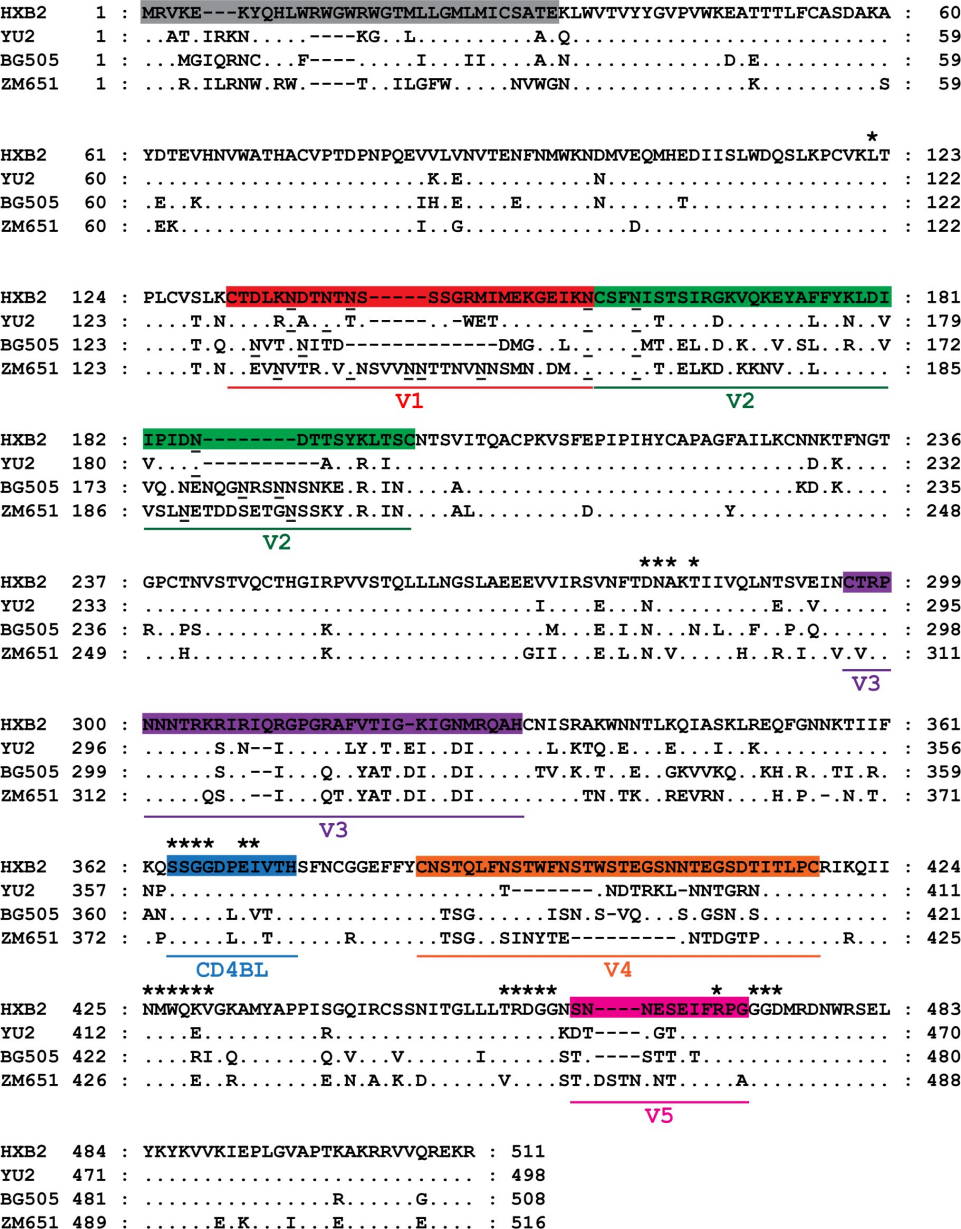
Amino acid sequence alignment of the gp120 subunit for HIV-1_HXB2_, HIV-1_YU2_, HIV-1_BG505_, and HIV-1_ZM651_. The amino acid sequences of the signal peptide (grey), V1 (red), V2 (green), V3 (purple), V4 (orange), and V5 (pink) domains and the CD4 binding loop (CD4BL; blue) are indicated. The periods (.) indicate residues of HIV-1_YU2_, HIV-1_BG505_, and HIV-1_ZM651_ that are identical to HIV-1_HXB2_. The asterisks (*) denote the 26 residues of the gp120 subunit that directly contact CD4 [3]. Gaps in the sequence are indicated by a hyphen (-). The underlined asparagines in the V1 and V2 domains indicate the potential N-linked glycosylations predicted by N-Glycosite [42]. The numbers flanking the sequences are the position numbers of the residues for the isolate.

The differences in either amino acid sequence or secondary structures of the gp120 subunit from the HIV-1_BG505_ and HIV-1_ZM651_ isolates compared to HIV-1_HXB2_ and HIV-1_YU2_ might contribute to the differences in eCD4-Ig^mim2^ sensitivity. A comparison of the CD4BL identified a proline at position 369 and valine at position 372 (HIV-1_HXB2_ numbering) present within the CD4BL of Env from HIV-1_HXB2_ and HIV-1_YU2_, while a leucine and threonine were present at the same respective positions in HIV-1_BG505_ and HIV-1_ZM651_ (Fig 4).

**Fig 5:**
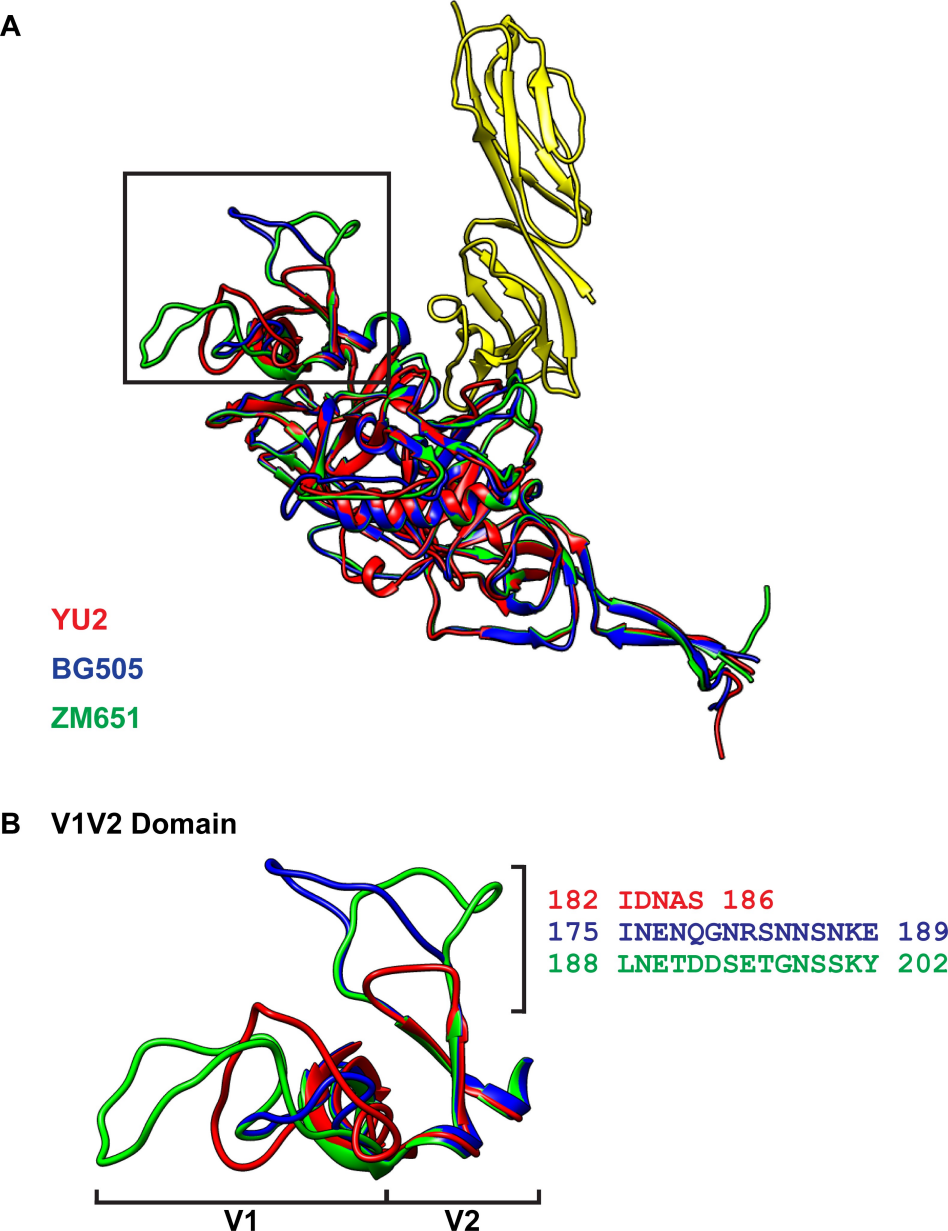
Comparison of the V1V2 domain of structural homology models of HIV-1_YU2_, HIV-1_BG505_, and HIV-1_ZM651_. (A) Ribbon diagrams of predicted models of full length gp120 of HIV-1_YU2_ (red), HIV-1_BG505_ (blue), and HIV-1_ZM651_ (green) generated by RaptorX and compared using Matchmaker (UCSF Chimera). The CD4 domains 1 and 2 (2QAD) are indicated in yellow. The boxed area highlights the V1V2 domains of the models and is magnified in (B). The right hand bracket indicates a loop region of V2 that is 10 amino acids longer in HIV-1_BG505_ and HIV-1_ZM651_ than HIV-1_YU2_. The amino acid sequences of the loop for the isolates are represented with their respective position numbers. The V1 and V2 domains are indicated by the lower brackets.

The length of the V1 and V2 domains of gp120 and the number of potential N-linked glycosylations within the domains have also been demonstrated to be a contributing factor to protect HIV-1 from CD4BS directed antibodies, suggesting that the V1 and V2 domains might occlude the CD4BS [58]. Among the three CCR5 isolates evaluated, both HIV-1_BG505_ and HIV-1_ZM651_ gp120 subunits had longer V1 and V2 domains of 69 and 82 aa, respectively, than HIV-1_YU2,_ which was 66 aa in length (Fig 4; Table 2). Because of the limited structure data on the V1 and V2 domain, predicted models were generated for the gp120 subunits of HIV-1_YU2_, HIV-1_BG505_ and HIV-1_ZM65_. The gp120 subunit V2 domains of HIV-1_BG505_ and HIV-1_ZM651_ were 10 aa longer than HIV-1_YU2_ (Fig 5). In addition, the gp120 subunits of HIV-1_BG505_ and HIV-1_ZM651_ were predicted to have six and nine potential N-linked glycosylated asparagines in the V1 and V2 domains, respectively, in contrast to HIV-1_YU2_ that only had five. The longer V2 domain and greater number of potential N-linked glycosylations in the gp120 subunit of HIV-1_BG505_ and HIV-1_ZM651_ might contribute to their reduced eCD4-Ig^mim2^ sensitivity, possibly by limiting access of CD4 domains 1 and 2 of the inhibitor to the CD4BS.

**Table 2:** The number of residues and predicted N-glycosylation (PNG) sites in the V1 and V2 domains of the HIV isolates.

Virus	V1		V2		V1V2 Total	
	Residues	PNG	Residues	PNG	Residues	PNG
**HXB2**	26	3	40	2	66	5
**YU2**	25	3	38	2	63	5
**BG505**	18	3	48	3	66	6
**ZM651**	31	6	48	3	79	9

## Discussion

In this study, a modified SRFA adapted to model HIV Env fusion was evaluated using neutralizing and non-neutralizing gp120-binding antibodies. Both the 2G12 and VRC02 mAbs were able to inhibit HIV-1_HXB2_ Env fusion in the SRFA, which was consistent with previously reported neutralization data. While 2G12 has been proposed to interfere with binding of the gp120 subunit V3 domain and CCR5, the reduced fusion indicates that this inhibition is unlikely to be the sole mechanism of action of 2G12 because HIV-1_HXB2_ can infect cells that do not express CCR5 [59,60]. The reduction in HIV-1_HXB2_ Env fusion by VRC03 is likely a result of the antibody masking the CD4BS, preventing the binding of CD4 to gp120. In contrast, HIV-1_HXB2_ Env fusion was not inhibited by the CD4i epitope binding mAbs, 17b and 48d, or the 697-30D mAb. The lack of HIV-1_HXB2_ Env fusion inhibition by both the CD4i mAbs supports past conclusions that the epitope which includes the chemokine-receptor binding site/V3 domain is not readily accessible to the mAb until the gp120 subunit interacts with CD4 [3,53]. This requirement would limit the opportunity for both mAbs to bind to gp120 and interfere directly or indirectly with fusion. The inability of 697-30D mAb to inhibit fusion in the SRFA was consistent with reports that it is only able to neutralize primary isolates [26]. Thus, the correlation of fusion inhibition with previously reported data of the antibodies evaluated demonstrated that the modified SRFA models Env fusion.

The reduced sensitivity of HIV-1_BG505_ and HIV-1_ZM651_ to fusion inhibition and neutralization by eCD4-Ig^mim2^ correlated with differences in sequence and length of the gp120 V1 and V2 domains compared to HIV-1_HXB2_ and HIV-1_YU2_. The correlation of low eCD4-Ig^mim2^ sensitivity with a longer V2 domain and increase in the number of potential N-glycosylation sites in the V1 and V2 domains of HIV-1_BG505_ and HIV-1_ZM651_ supports past conclusions that the V1 and V2 domains can protect HIV-1 against CD4BL directed antibodies [58]. The eCD4-Ig^mim2^ sensitivity could be dependent on a single difference or a combination of the differences identified.

In conclusion, HIV Env fusion can be modeled by the SRFA and used to screen antibodies and neutralizing molecules for fusion-inhibition properties, whether these are direct or indirect. Sensitivity to the eCD4-Ig^mim2^ fusion inhibition was found to differ among HIV isolates using the SRFA developed in this study. The data can be used as a platform to improve the eCD4-Ig^mim2^ potency against isolates that are more resistant to eCD4-Ig^mim2^ neutralization.
